# Physiological and Functional Effects of Dominant Active TCRα Expression in Transgenic Mice

**DOI:** 10.3390/ijms24076527

**Published:** 2023-03-30

**Authors:** Anastasiia A. Kalinina, Rustam Kh. Ziganshin, Yulia Yu. Silaeva, Nina I. Sharova, Margarita F. Nikonova, Nadezda A. Persiyantseva, Tatiana G. Gorkova, Elena E. Antoshina, Lubov S. Trukhanova, Almira D. Donetskova, Victoria V. Komogorova, Marina M. Litvina, Alexander N. Mitin, Maria A. Zamkova, Alexandra V. Bruter, Ludmila M. Khromykh, Dmitry B. Kazansky

**Affiliations:** 1 N.N. Blokhin National Medical Research Center of Oncology, Ministry of Health of the Russian Federation, Kashirskoe sh., 24, 115478 Moscow, Russia; 2 Shemyakin-Ovchinnikov Institute of Bioorganic Chemistry, Russian Academy of Sciences, Miklukho-Maklaya st. 16/10, 117997 Moscow, Russia; 3 Institute of Gene Biology, Russian Academy of Sciences, Vavilova st. 34/5, 119334 Moscow, Russia; 4 National Research Center, Institute of Immunology Federal Medical-Biological Agency of Russia, Kashirskoe sh., 24, 115522 Moscow, Russia; 5 Center for Precision Genome Editing and Genetic Technologies for Biomedicine, Institute of Gene Biology, Russian Academy of Sciences, 34/5 Vavilov St., 119334 Moscow, Russia

**Keywords:** chain-centric TCR, dominant active TCRα, allogeneic immune response, thymic involution, TCR repertoire, proteomics, aging, transgenesis, transgenic mice

## Abstract

A T cell receptor (TCR) consists of α- and β-chains. Accumulating evidence suggests that some TCRs possess chain centricity, i.e., either of the hemi-chains can dominate in antigen recognition and dictate the TCR’s specificity. The introduction of TCRα/β into naive lymphocytes generates antigen-specific T cells that are ready to perform their functions. Transgenesis of the dominant active TCRα creates transgenic animals with improved anti-tumor immune control, and adoptive immunotherapy with TCRα-transduced T cells provides resistance to infections. However, the potential detrimental effects of the dominant hemi-chain TCR’s expression in transgenic animals have not been well investigated. Here, we analyzed, in detail, the functional status of the immune system of recently generated 1D1a transgenic mice expressing the dominant active TCRα specific to the H2-K^b^ molecule. In their age dynamics, neither autoimmunity due to the random pairing of transgenic TCRα with endogenous TCRβ variants nor significant disturbances in systemic homeostasis were detected in these mice. Although the specific immune response was considerably enhanced in 1D1a mice, responses to third-party alloantigens were not compromised, indicating that the expression of dominant active TCRα did not limit immune reactivity in transgenic mice. Our data suggest that TCRα transgene expression could delay thymic involution and maintain TCRβ repertoire diversity in old transgenic mice. The detected changes in the systemic homeostasis in 1D1a transgenic mice, which are minor and primarily transient, may indicate variations in the ontogeny of wild-type and transgenic mouse lines.

## 1. Introduction

A T cell receptor (TCR) is a heterodimer consisting of α- and β-chains. The classical model states that there is an equal contribution of both TCR hemi-chains in the recognition of peptide-MHC complexes (pMHC) [1]. Accumulating evidence, however, suggests that this is not the case for all TCRs. Structural analyses showed that TCR α-chain (TCRα) can dictate the orientation of the receptor on a pMHC complex and be in more contact with it [2]. Furthermore, the α-chain can alter how the TCR β-chain interacts with pMHC and change the TCR’s overall specificity [3]. Several studies have described TCRs with a dominant α-chain [3,4,5,6,7]. The predominant role of the TCR β-chain in antigen recognition was also pointed out [8,9,10]. Bouneaud et al. used TCRβ transgenic mice to show that the β-chain can dictate T cell avidity, TCRα structure, and guide the process of negative selection [10]. Ochi et al. characterized the β-chain that dictates TCR specificity, while its pairing with various α-chains influenced receptor avidity [8]. Certain public β-chains were found to control TCR’s reactivity in studies of both public and private TCRβ variants [9]. In 2015, the research group led by N. Hirano introduced the term “chain-centric” TCR to characterize a receptor in which either α- or β-chains dominate in antigen recognition [7,8].

Our recent studies revealed that the TCR repertoire of memory T cells regularly contains chain-centric TCRs, and nearly 20% of these receptors have a dominant active α-chain [5]. We proved that such a dominant active α-chain can recognize tumor or bacterial antigens both in vitro and in vivo [4,5,11]. In tumor and infectious mouse models, our findings demonstrated that the adoptive transfer of naive T cells transduced with an individual dominant active TCRα provided a therapeutic benefit to a syngeneic recipient [4,5,11].

To better understand the role of dominant active TCRα in tumor antigen recognition, we generated transgenic mice expressing the α-chain of the chain-centric TCR specific to the MHC I class molecule H-2K^b^ [4]. These transgenic 1D1a mice accelerated the rejection of EL-4 lymphoma carrying the specific antigen, and the dynamics of EL-4 elimination were 2.0 times faster than they were in wild-type animals and comparable to those in pre-immunized control mice with the established specific pool of memory T cells [4]. Our data showed that 1D1a mice had an inborn pre-formed pool of CD8+ effector and memory anti-EL-4 T cells due to the expression of a single dominant active TCRα specific to the H-2K^b^ molecule [4].

These findings open up a great perspective for the generation of transgenic animals with T cells expressing individual dominant active α-chains of chain-centric TCRs. These transgenic animals might display enhanced anti-tumor immune control or inheritable resistance to infections. Additionally, since mature peripheral T cells can express two α-chains coupled with one β-chain, transgenesis of the single α-chain could increase the diversity of the TCR repertoire because α-chains are not subjected to allelic exclusion during thymocyte development [12]. Hence, it is hypothesized that TCR repertoire expansion will improve the host’s immune response, especially in aging organisms, when TCR repertoire contraction is known to occur [13].

However, random pairing of the transgenic α-chain with endogenously expressed β-chains can potentially generate autoreactive TCRs. Alternatively, transgenic α-chain expression could change the TCR repertoire, which may affect the homeostasis and specificity of peripheral T cells. The entire organism of a transgenic animal, as well as the immune system’s homeostasis and functional activity, may be affected by the expression of the dominant active TCRα. To address these issues, we used previously generated 1D1a transgenic mice to assess the physiological and functional effects of the dominant active TCRα expression. Age-related changes in transgenic and wild-type mice at the ages of 3, 6, and 12 months were investigated.

## 2. Results

### 2.1. Dominant Active TCRα Expression Affects the Homeostasis of Lymphoid Organs in 1D1a Transgenic Mice

**Figure 1:**
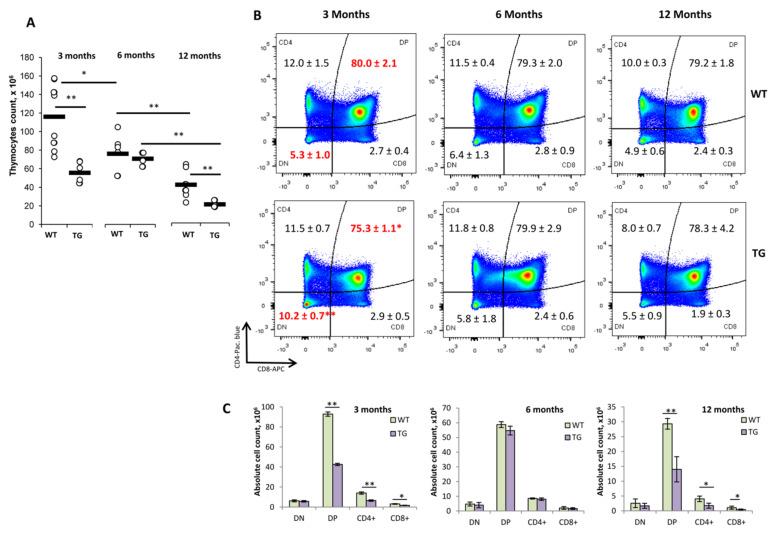
The figure was changed accordingly. Effects of the dominant active TCRα expression on the thymus homeostasis of 1D1a transgenic mice. Wild-type B10.D2(R101) (WT) and transgenic 1D1a (TG) mice at the ages of 3, 6, and 12 months (3 Mo, 6 Mo, and 12 Mo, respectively) were used to analyze the absolute thymocyte count (×10^6^ per thymus) (**A**) and the proportion (%) of main thymocyte subpopulations as determined by the expression of CD4 and CD8 markers by flow cytometry (**B**). The absolute counts (×10^6^) of double-negative (CD4−CD8−, DN), double-positive (CD4+CD8+, DP), and single-positive CD4+ and CD8+ thymocytes (**C**) in the thymus of 3 Mo (left panel), 6 Mo (middle panel), and 12 Mo (right panel) WT and TG mice were then calculated. Data are presented as mean ± SEM (n = 6–10). * *p* < 0.05; ** *p* < 0.01 (unpaired Student *t*-test).

**Figure 2:**
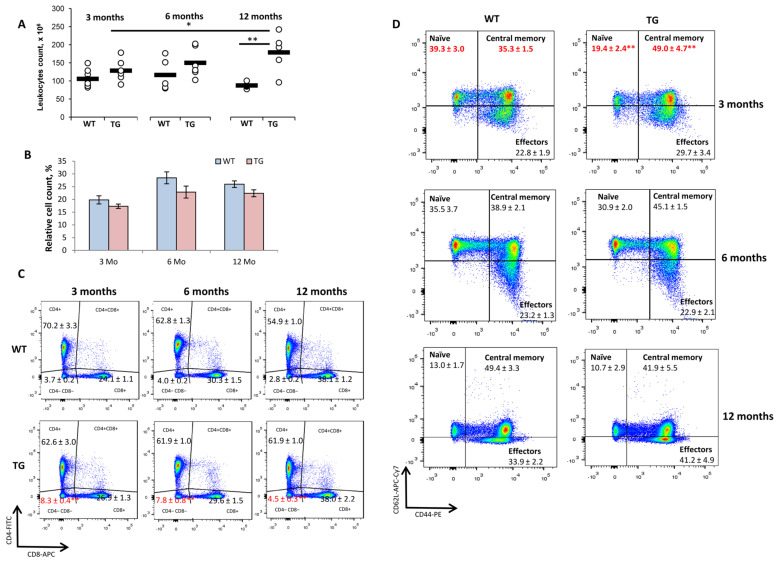
Effects of the dominant active TCRα expression on the spleen homeostasis of 1D1a transgenic mice. The spleens of wild-type B10.D2(R101) (WT) and transgenic 1D1a (TG) mice at the ages of 3, 6, and 12 months (3 Mo, 6 Mo, and 12 Mo, respectively) were isolated and analyzed. (**A**) The total leukocyte count (×10^6^ per spleen). (**B**–**D**) Flow cytometry analyses of spleen T cell populations in WT and TG mice. (**B**) The proportion (%) of T cells (CD3+). (**C**) The proportion (%) of CD4+, CD8+, and double-negative CD4−CD8− T cells. (**D**) The proportion (%) of CD8+ T cells with the phenotype of naïve cells (CD62L+CD44−), central memory cells (CD62L+CD44+), and effectors (CD62L−CD44+). Data are presented as mean ± SEM (n = 5–10). * *p* < 0.05; ** *p* < 0.01 (unpaired Student *t*-test).

To evaluate possible effects of the transgenic TCR α-chain expression on the homeostasis of the thymus and spleens of 1D1a transgenic mice, we analyzed age-related subpopulation changes in these organs in 3–12-month-old wild-type B10.D2(R101) (WT) and transgenic 1D1a(TG) mice (Figure 1 and Figure 2).

The absolute cell count in the thymus of 3-month-old (3 Mo) TG mice was two-fold decreased compared to that of the aged-matched WT control mice (Figure 1A). The thymus is known to involute during aging. As expected, the thymocyte counts in 6 Mo and 12 Mo WT mice were 1.5- and 3.1-fold decreased compared to that of 3 Mo WT mice, respectively (Figure 1A). In TG mice, the absolute cell count in the thymus of 3 Mo and 6 Mo mice was comparable, and only at 12 months, it decreased 3.8-fold compared to that of the younger TG mice (Figure 1A). The thymocyte count of 12 Mo TG mice was two-fold lower compared to that of the age-matched WT control mice (Figure 1A). That could be due to initially smaller thymus cell counts in TG mice.

Consistent with our previous studies [4], in 3-month-old TG mice compared to the respective WT control, there was a significant increase in the proportion of double-negative (CD4−CD8−, DN) cells and a decrease in the number of double positive (CD4+CD8+, DP) cells (Figure 1B). In terms of the absolute cell count, however, the number of DN thymocytes in 3 Mo TG was comparable to that of the control, while the numbers of DP and single-positive (SP) CD4+ and CD8+ thymocytes were decreased nearly two-fold compared to those of age-matched WT mice (Figure 1C, left panel). These data could point out changes in intrathymic T cell development due to the expression of pre-formed transgenic TCRα [4]. No changes in the main subpopulations of thymocytes in the absolute and relative cell counts were detected in 6 Mo TG mice (Figure 1B,C, middle panel). In 12 Mo TG mice, although the proportions of the main thymocyte subpopulations were not altered (Figure 1B), the absolute counts of DP and SP thymocytes (similar to those of 3 Mo TG mice) were approximately two-fold reduced compared to those of the age-matched WT controls (Figure 1C, right panel). This effect could result from the twice lower total thymocyte count in aged 1D1a mice compared to that of the control mice (Figure 1A).

The leukocyte counts in the spleens of WT and TG mice were comparable at the ages of 3 and 6 months. Surprisingly, the splenocyte count in 12-month-old TG mice was 2.2-fold higher than it was in the age-matched WT control and 1.4-fold higher than it was in the 3 Mo TG mice (Figure 2A). This could suggest an autoimmune lymphoproliferative syndrome in aged transgenic mice because of dominant active TCRα expression. However, at all of the ages studied, the background levels of splenocytes in vitro proliferation were comparable in WT and TG mice (Supplementary Figure S1A). Furthermore, in 12-month-old mice, the proportion of T cells (Figure 2B), B cells, monocytes/macrophages, and neutrophils (Supplementary Figure S1B) was not changed compared to that of the age-matched WT control, which is indicative of no biased cell accumulation.

The CD3+ percentage and the CD4/CD8 T cell ratio were not affected in TG mice of any age compared to that of the respective control WT mice (Figure 2B,C). However, we detected 2.2-, 1.9-, and 1.8-fold increases in the proportion of DN CD4−CD8− T lymphocytes in the spleens of 3, 6, and 12 Mo TG mice, respectively, compared to that of the age-matched WT mice (Figure 2C). The proportions of TCRγδ T cells within CD3+ DN spleen cells were comparable in WT and TG mice and accounted for 50–60% (Supplementary Figure S1C), indicating that DN T cells also contained TCRαβ+ T lymphocytes.

As previously described [4], the population of central memory CD8+ T cells (CD62L^hi^CD44^hi^) was significantly increased and the amount of naive CD8+ cells (CD62L^hi^CD44^low^) was decreased in the spleens of 3-month-old TG mice compared to those of the WT control mice (Figure 2D, upper panel). These changes were not observed in 6–12-month-old TG mice (Figure 2D, middle and bottom panels, respectively).

### 2.2. TCRα/β Repertoire of Peripheral T Cells Is Moderately Reshaped by Transgenic Dominant Active TCRα Expression 

**Figure 3:**
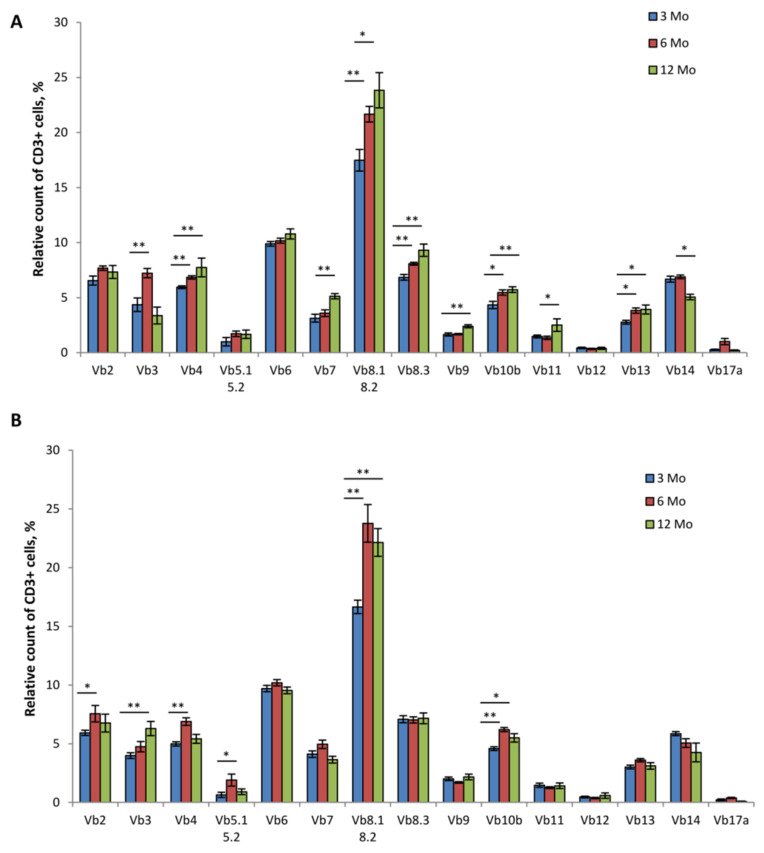
Changes in the TCRβ repertoire of peripheral T cells in 1D1a transgenic mice with the age-dependent dynamics. Leukocytes were isolated from the blood of wild-type B10.D2(R101) (WT) and transgenic 1D1a (TG) mice at the ages of 3, 6, and 12 months (3 Mo, 6 Mo, and 12 Mo, respectively). The proportion (%) of CD3+ cells expressing different TCRVβ families was evaluated by flow cytometry in the pool of peripheral leukocytes of WT (**A**) and TG (**B**) mice. Data are presented as mean ± SEM (n = 5–6). * *p* < 0.05; ** *p* < 0.01 (Kruskal–Wallis test).

To evaluate the effects of dominant active TCR α-chain expression on the diversity of TCR β-chain repertoire in transgenic 1D1a mice in terms of age dynamics, we analyzed the percentage of peripheral T cells that expressed β-chains belonging to different Vb families. The peripheral repertoires of TCR β-chains of the age-matched WT mice were used for the comparison (Figure 3).

Our data showed that in 6- and 12-month-old WT mice, the TCRβ repertoire underwent alterations compared to that of the 3-month-old WT mice (Figure 3A). The percentages of T cells with β-chains from six Vb families (Vb3, Vb4, Vb8.1/8.2, Vb8.3, Vb10b, and Vb13) were higher in 6-month-old WT mice compared to those of the 3-month-old WT animals (Figure 3A). In 12-month-old WT mice, the counts of T lymphocytes with β-chains of eight Vb families (Vb4, Vb7, Vb8.1/8.2, Vb8.3, Vb9, Vb10b, Vb11, and Vb13) were further increased compared to the respective counts in 3 Mo WT mice (Figure 3A). This was accompanied by a significant drop of Vb14+ T cells in 12 Mo WT mice (*p* < 0.05; Figure 3A).

In TG mice, age-related changes in the TCRβ repertoire were less marked (Figure 3B). We detected a transitory increase in the proportion of T cells with β-chains from Vb2, Vb4, Vb5.1/5.2, Vb8.1/8.2, and Vb10b families in 6-month-old TG mice that exceeded the corresponding cell count in 3-month-old TG mice by 1.3-, 1.4-, 2.9-, 1.4-, and 1.3-fold, respectively (Figure 3B). These changes were not observed in 12 Mo TG mice. However, in these mice, the percentages of Vb3+, Vb8.1/8.2+, and Vb10b+ T cells were 1.6-, 1.3-, and 1.2-fold higher, respectively, compared to that of the 3 Mo TG mice (*p* < 0.05; Figure 3B).

**Figure 4:**
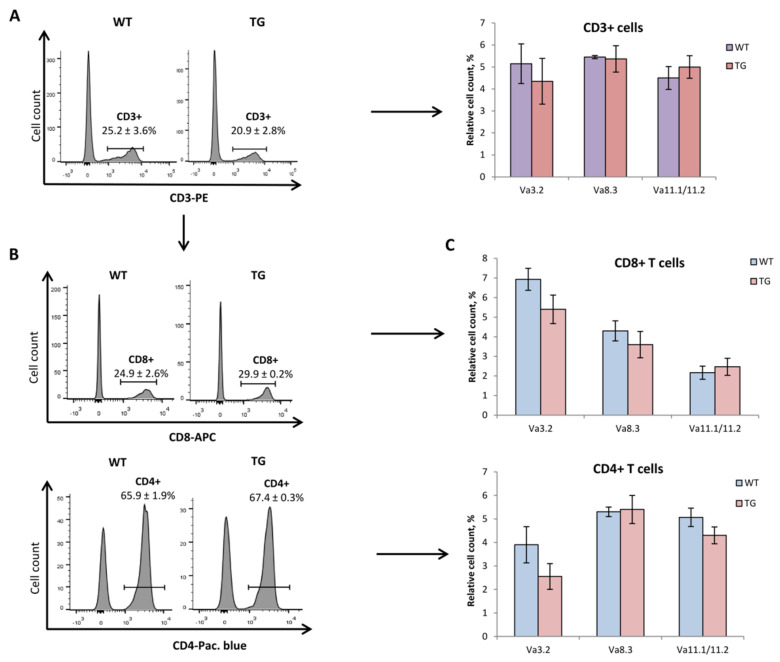
Analysis of the TCRα repertoire of peripheral T cells in 1D1a transgenic mice. Leukocytes were isolated from the blood of wild-type B10.D2(R101) (WT) and transgenic 1D1a (TG) mice at the age of 3 months. (**A**) The proportion (%) of T cells expressing different TCRVα families was evaluated by flow cytometry in the pool of peripheral CD3+ leukocytes. (**B**) The proportions (%) of CD4+ and CD8+ cells were assessed in the pool of peripheral CD3+ leukocytes of WT and TG mice. (**C**) The proportion (%) of T cells with endogenous TCRVα families was analyzed individually for CD4+ and CD8+ subsets of peripheral CD3+ cells of WT and TG mice. Data are presented as mean ± SEM (n = 4).

Our recent data showed that the T cell counts with various β-chains were comparable in 3-month-old WT and TG mice [4] (Figure 3A,B), indicating no preferable pairing of the transgenic α-chain with its endogenously expressed counterparts. This was confirmed in the study here (Supplementary Figure S2, upper panel). Furthermore, we compared the proportions of T cells with different β-chains individually in populations of CD4+ and CD8+ peripheral T cells of young TG mice and found no significant differences compared to that of the age-matched control (Supplementary Figure S3). Consistent with the analysis of the spleens (Figure 2C), the CD4/CD8 ratio of peripheral blood T lymphocytes was also not altered in 1D1a mice (Figure 4), confirming no bias of the transgenic α-chain expression in these T cell subsets.

Next, we analyzed the percentage of T cells (CD3+) with various β-chains in 6 Mo and 12 Mo TG and WT mice (Supplementary Figure S2, middle and lower panels, respectively). In 6 Mo TG mice, T cells expressing β-chains of the Vb7 and Vb10b families were 1.4- and 1.2-fold higher, while T cells with β-chains of the Vb3 and Vb14 families were 1.5- and 1.4-fold lower compared to those of the WT control (Supplementary Figure S2, middle panel). In 12 Mo TG mice, the proportion of Vb3+ T cells was 1.8-fold higher compared to that of the age-matched WT mice, while the numbers of Vb4+ and Vb8.3+ T cells were 1.4- and 1.3-fold lower (Supplementary Figure S2, lower panel).

Since the rearrangement of α-chains is not restricted by allelic exclusion, expression of transgenic TCRα does not interfere with the rearrangements of endogenous α-chains. To prove that dominant active TCRα expression does not disturb the repertoire of endogenous α-chains in 1D1a mice, we evaluated the percentages of T cells displaying endogenous α-chains that belong to the Va3.2, Va8.3, and Va11.1/11.2 families in the blood of 3 Mo TG animals (notably, the transgenic 1D1α-chain belongs to the Va11.3 family, for which there are no commercial antibodies [4]) (Figure 4). The percentages of T cells expressing these TCRα variations were not changed in 1D1a transgenic mice compared to those of the age-matched WT animals, as shown by analyses of the total population of CD3+ cells (Figure 4A) and individually for CD4+ and CD8+ T cell subsets (Figure 4B,C).

### 2.3. Histological Evaluations of Lymphoid and Non-Lymphoid Organs of Transgenic 1D1a Mice Revealed No Pathomorphological Changes 

The pairing of the transgenic α-chain with endogenously expressed β-chains could potentially create autoreactive TCRs. However, during intrathymic development, autoreactive clones must be deleted by negative selection or differentiate into thymic regulatory T cells. To prove the low frequency of generation of autoreactive T cell clones expressing dominant active TCRα, we performed histological analysis of lymphoid (thymus, spleens, and lymph nodes) and non-lymphoid (lungs, hearts, livers, kidneys, thyroid glands, pancreas, adrenal glands, and small and large intestines) organs of 3–12-month-old TG and WT males (Supplementary Figure S4) and females. No pathological changes were detected in the viscera of the TG mice compared to the viscera of their respective WT controls (Supplementary Figure S4).

### 2.4. Dominant Active TCRα Expression Influences the Development of Specific and Non-Specific Immune Responses in Transgenic 1D1a Mice

To evaluate the effects of dominant active TCRα expression on the functional status of the immune system of transgenic 1D1a mice, levels of the immune response to the specific antigen (H-2K^b^) and third-party antigens (H-2^q^ alloantigens and sheep red blood cells (SRBCs)) were evaluated in TG mice aged 3–12 months.

**Figure 5:**
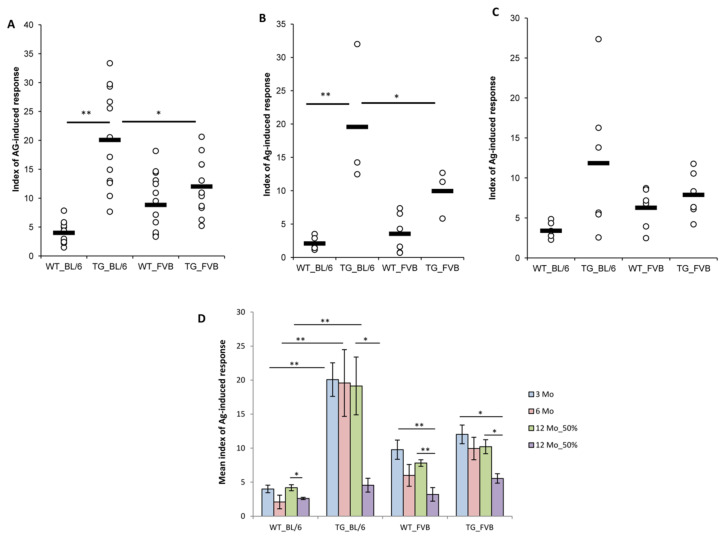
Effects of the dominant active TCRα expression on the development of an allogeneic immune response in 1D1a transgenic mice. Splenocytes from wild-type B10.D2(R101) (WT) and transgenic 1D1a (TG) mice were isolated and cultured in the mixed lymphocyte reaction with mitomycin C-treated splenocytes of C57BL6 (BL/6) or FVB mice for 72 h. The level of responder cells proliferation was measured by ^3^H-thymidine incorporation. Indices of the antigen-induced immune response were calculated as described in the Section 4 for 3-month-old (**A**), 6-month-old (**B**), and 12-month-old (**C**) WT and TG mice. (**D**) The mean indices of the immune response to the specific (BL/6) and third-party (FVB) alloantigens in WT and TG mice aged 3–12 months (3 Mo, 6 Mo, and 12 Mo). In aged (12 Mo) WT and TG mice, two distinct but equal groups of animals were detected: those with unchanged or decreased antigenic responses. Thus, for 12 Mo mice, the mean response indices were calculated individually for the indicated groups of animals. Data are presented as mean ± SEM (n = 3–12). * *p* < 0.05; ** *p* < 0.01 (Kruskal–Wallis test and Mann–Whitney U-test).

As previously described, non-immunized (intact) transgenic 1D1a mice exhibited a strong immune response to EL-4 and C57BL/6 mice, both expressing the specific antigen (H-2K^b^) [4]. The level of immune response to the specific antigen (anti-C57BL/6) in mixed lymphocyte reaction in vitro was five-fold higher in intact TG mice at the ages of 3 and 6 months compared to that of the age-matched intact WT mice (Figure 5A,B). In 12 Mo TG mice, one half of the animals responded to the C57BL/6 alloantigen similarly to the younger mice (Figure 5C,D), but 50% of the aged TG mice had a four-fold decreased immune response to H-2K^b^, a level that was comparable to the immune response of 3–6-month-old WT mice (Figure 5C,D). However, the observed effect could not be attributed to the transgenic α-chain expression because 50% of the aged (12-month-old) WT mice also had a decreased immune response to the C57BL/6 antigen (Figure 5C,D).

The levels of the in vitro immune response to FVB, expressing third-party alloantigens (H-2^q^), were comparable in TG and WT mice of all the ages studied (Figure 5). Again, we detected a significant two-fold decrease in the response to FVB in 50% of 12-month-old TG and WT mice compared to that of respective younger (3 and 6 Mo) mice (Figure 5D). This could presumably be due to age-related TCRβ repertoire contraction (Figure 3) [13]. Our results here are in line with an earlier study that suggested that a decrease in the diversity of the TCR repertoire may reduce immunological function in elderly individuals [14].

**Figure 6:**
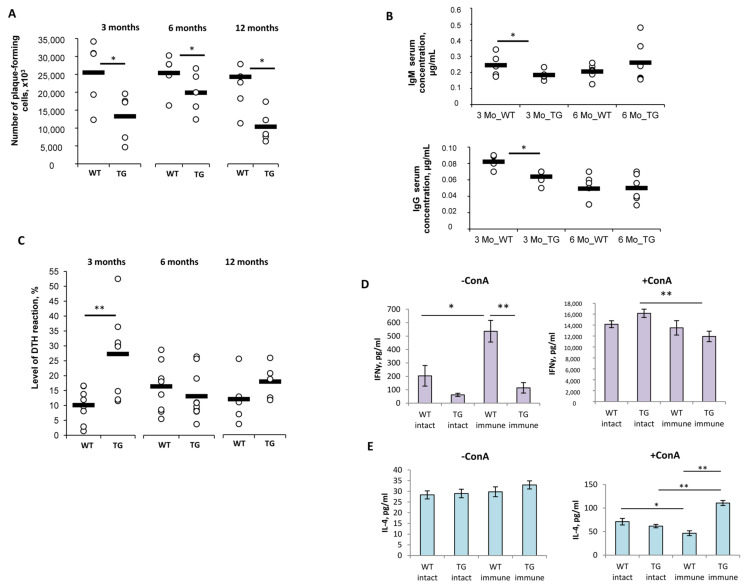
Effects of the dominant active TCRα expression on the development of the humoral immune response and the delayed-type hypersensitivity reaction in transgenic 1D1a mice. Wild-type B10.D2(R101) (WT) and transgenic 1D1a (TG) female mice at the ages of 3, 6, and 12 months (3 Mo, 6 Mo, and 12 Mo, respectively) were immunized with sheep red blood cells (SRBCs). (**A**) The number of plaque-forming cells (×10^3^) in the spleens of mice was counted on day 5 post-immunization. (**B**) Immunoglobulin levels (μg/mL) in the blood serum of non-immunized (intact) 3- and 6-month-old WT and TG female mice were measured by ELISA. (**C**) The level of the delayed-type hypersensitivity (DTH) reaction (%) in mice was assessed 24 h after injection of the SRBCs resolving dose. To evaluate cytokine production, WT and TG female mice (n = 3) were immunized with SRBCs, and three days later, their splenocytes were isolated and cultured for 24 h with 3 μg/mL of concanavalin A (+ConA) or without stimulation (-ConA). Levels of IFN-γ (**D**) and IL-4 (**E**) production in cell cultures were measured by ELISA. Data are presented as mean ± SEM. * *p* < 0.05; ** *p* < 0.01 (unpaired Student *t*-test and Mann–Whitney U-test).

Analysis of the humoral immune response to SRBCs showed its suppression in TG mice of all the ages studied. The numbers of plaque-forming cells in the spleen were 2.2-, 1.4-, and 2.8-fold decreased in TG mice at 3, 6, and 12 Mo, respectively, compared to those of the age-matched WT mice (Figure 6A). To test if this effect could be due to the immunodeficiency developed in TG mice, immunoglobulin serum concentrations were measured in 3- and 6-month-old TG and WT mice (Figure 6B). The levels of both IgM and IgG were significantly (*p* < 0.05) decreased in 3 Mo TG mice compared to that of the WT control mice (Figure 6B). These changes were not detected in 6 Mo TG mice (Figure 6B).

In contrast to the humoral response, the delayed-type hypersensitivity (DTH) reaction to SRBCs was 2.7-fold more intensive in 3-month-old TG mice compared to that of the age-matched WT control mice (Figure 6C). However, in 6- and 12-month-old TG mice, the level of DTH was comparable to that of the respective WT control mice (Figure 6C).

The observed discrepancy in the humoral response and DTH reaction to the model antigen in 3-month-old TG mice (Figure 6A,C) could probably indicate the imbalance of Th1 and Th2 cytokines in these mice. To test this hypothesis, 3-month-old TG and WT mice were immunized with SRBCs, and three days later, their splenocytes were isolated and cultured for 24 h with or without concanavalin A (ConA) stimulation. The levels of IFNγ and IL-4 were subsequently measured in culture mediums by ELISA (Figure 6D,E).

No significant differences in IFNγ production were detected in the cultures of non-stimulated cells from both TG and WT non-immunized (intact) mice (Figure 6D). As expected, non-stimulated splenocytes of immunized WT mice produced 2.6-fold higher levels of IFNγ compared to those of the intact WT mice. Interestingly, this effect was not observed in TG mice, and IFNγ production in non-stimulated cells of immunized TG mice was the same as it was in intact TG mice (Figure 6D, left panel). Furthermore, the ConA stimulation of splenocytes from immunized TG mice led to a significant drop in IFNγ production compared to that of the stimulated cells from intact TG mice (Figure 6D, right panel). In the culture of ConA-stimulated cells from both intact and immunized WT mice, the IFNγ levels were comparable.

The levels of IL-4 production in the non-stimulated cell cultures of both intact and immunized WT and TG mice were comparable (Figure 6E, left panel). The ConA stimulation of splenocytes from immunized WT mice decreased IL-4 production by 1.5-fold compared to that of then stimulated cells from intact WT mice (Figure 6E, right panel). Notably, the ConA stimulation of splenocytes from TG mice had the opposite effect, stimulating IL-4 production in the cell culture of immunized TG mice by 1.8-fold compared to that of the intact TG mice and by 2.4-fold compared to that of the immunized WT mice (Figure 6E, right panel).

### 2.5. Blood Tests of 1D1a Transgenic Mice Are Not Affected by the Dominant Active TCRα Expression

To analyze possible effects of the dominant active TCRα expression on the systemic homeostasis of transgenic 1D1a mice, blood tests were performed in 3–12-month-old TG and WT mice. The blood counts of TG mice of all ages studied were comparable to those of the age-matched WT mice (Supplementary Table S1). All but one blood biochemical parameter of TG animals did not differ from the respective WT control (Supplementary Table S2). We detected a 1.4-fold increased content of alkaline phosphatase in the blood of 3-month-old TG mice compared to that of the age-matched WT mice (Supplementary Table S2). This difference was not observed in 6 Mo and 12 Mo mice.

### 2.6. Plasma Proteome Analysis Revealed Sex- and Age-Dependent Changes in Transgenic 1D1a Mice

For more detailed evaluation of the homeostasis in TG mice, we performed comparative proteomic analysis of the blood plasma of 3–12-month-old TG and WT animals. For this, we used an experimental protocol that allowed us to compare the contents of plasma proteins without the preliminary removal of major plasma proteins. To broaden the list of identified and quantified minor plasma proteins, we created a spectral matching library using LC-MS/MS data of SCX fractions of tryptic hydrolysates of two plasma samples, in which the content of minor proteins was enriched by Proteominer technology.

**Figure 7:**
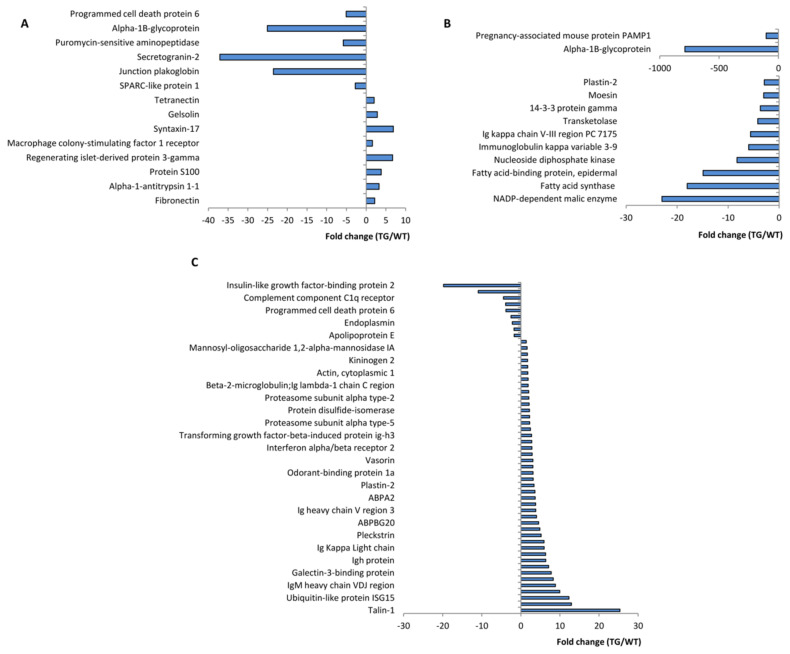
Proteomics analysis of the blood plasma proteins of transgenic 1D1a mice. The blood plasma of female and male wild-type B10.D2(R101) (WT) and transgenic 1D1a (TG) mice at the ages of 3, 6, and 12 months was subjected to chromatography-mass spectrometry analysis. Each sex and age group contained three animals. Comparative bioinformatics analyses were performed pair-wise for WT and TG of each sex and age group using the Perseus platform by MaxQuant. Data on significant differences in the plasma proteome between each sex and age group of WT and TG mice are presented as the fold change of the protein level in the plasma of TG mice relative to the respective level in the plasma of WT mice. (**A**) Proteome changes in the blood plasma of TG females at the age of 3 months. (**B**) Proteome changes in the blood plasma of TG females at the age of 12 months. (**C**) Proteome changes in the blood plasma of TG males at the age of 3 months.

Bioinformatics analysis of the mass spectrometry data revealed sex- and age-dependent changes in the content of some plasma proteins in TG mice relative to that of the WT controls (Figure 7). In female 1D1a mice at 3 months, the content of eight proteins was significantly increased, and the content of six proteins was decreased compared to that of the age-matched WT females (Figure 7A and Supplementary Table S3). No changes in the blood plasma proteomics were detected in TG female mice at the age of 6 months. In 12-month-old TG females, the relative content of 12 plasma proteins was significantly decreased (Figure 7B and Supplementary Table S3). Notably, changes in the plasma content of alpha-1B-glycoprotein, which were first observed in 3-month-old TG females, were sustained in aged (12-month-old) TG female mice (Supplementary Figure S5A). Moreover, we observed the age-dependent dynamics of its decline: the relative content of alpha-1B-glycoprotein was 25-fold decreased in 3 Mo TG females, while in 12 Mo TG females, it was decreased by nearly 800-fold compared to that of the respective age-matched control (Supplementary Figure S5A). Previous studies have shown that this protein is detected only in females, and its production increases with aging [15,16]. However, its functions remain largely unknown, and our data require further elucidation in this respect.

The most marked changes in plasma proteome were found in 3-month-old transgenic male mice (Figure 7C, Supplementary Table S3). The relative content of 48 proteins was significantly increased compared to that of the age-matched WT males (Figure 7C). These up-regulated plasma proteins belong to various functional clusters according to KEGG and GOTERM gene mapping (Supplementary Table S4). We also detected a significant decline in the content of 10 plasma proteins (Figure 7C) involved in the regulation of blood coagulation, triglyceride homeostasis, the regulation of synapse organization, and low-density lipoprotein particle receptor binding (Supplementary Table S4). However, these data must be interpreted with caution because changes in the functioning of individual cluster members cannot be conclusively explained as changes in the functioning of the entire cluster.

Interestingly, these dramatic changes in the plasma proteome were not observed in 6-month-old TG males, and the relative content of only one protein—the secreted form of uromodulin—was 50-fold decreased in the blood plasma of these TG males compared to that of the age-matched WT control (Supplementary Table S3). In 12-month-old transgenic male mice, the plasma proteome was also not significantly altered compared to that of the respective WT control. We found changes in only two plasma proteins in these TG mice: the relative content of alpha-fetoprotein was 8-fold lower, and the relative content of IgH protein was 2.6-fold higher in aged TG males compared to that of WT males (Supplementary Table S3). Notably, the increase in plasma IgH protein started in young (3-month-old) TG males when its content exceeded 7.1-fold higher than that of the respective WT control (Supplementary Figure S5B).

Comparative analysis of the plasma proteome of transgenic male and female mice showed that at the age of 3 months, three proteins were similarly altered in mice of both sexes (Supplementary Figure S5C). In TG males and females, the relative content of plasma programmed cell death protein 6 (PCDC6) was reduced by 3.8- and 5.0-fold, respectively. The content of SPARC-like protein 1 in the blood plasma of transgenic males and females was also decreased by 1.7- and 2.8-fold, respectively, compared to that of the WT controls (Supplementary Figure S5C). The relative gelsolin content increased by 1.7- and 2.8-fold in TG males and females, respectively. At the ages of 6 and 12 months, transgenic females and males did not share identical alterations in the plasma proteomics (Supplementary Table S3).

## 3. Discussion

The discovery of the chain-centricity phenomenon of some TCRs may have a substantial impact on TCR gene editing applications. For the generation of transgenic animals, the transgenesis of a single dominant active α-chain could produce animals with improved immunity without the significant alterations of the native TCR repertoire that occurs with the transgenesis of β-chains [17,18] or fully rearranged TCRs [19]. Our studies confirmed that this experimental approach can be used for the generation of transgenic animals with inheritable advanced anti-tumor immune defenses [4]. In the studies here, we evaluated the physiological and functional states of the immune system of previously generated 1D1a transgenic mice in terms of age dynamics to unveil the possible effects of dominant active TCRα expression on homeostasis and functioning of the immune system and whole organism.

Thymus analyses showed that 1D1a transgenic mice initially had thymocyte counts that had been reduced by twice as much as those of the wild-type control mice, but this was not accompanied by any pathomorphological changes in the organ (Supplementary Figure S4A), indicating normal thymus development. Thus, we assumed that a small thymus size in young 1D1a mice could be due to the expression of a fully formed transgenic TCR α-chain capable of accelerating intrathymic positive selection [4,20]. Notably, the transgenic α-chain expression in TCRβ+ double-negative (DN) thymocytes [21] had no adverse effect on T cell development in our experimental model, whereas several studies reported impaired thymic development due to transgenic α-chain expression that was forced non-physiologically early in DN thymocytes [21,22]. More importantly, our data suggested that the transgenic α-chain expression could slow thymus shrinkage, as it did not involute in 6-month-old TG mice (Figure 1A). Several mechanisms of thymic involution have been proposed, including TCR gene rearrangement blocking, deficiency of T cell progenitors, and modulation of the thymus microenvironment [23,24,25,26]. The findings from our transgenic 1D1a mouse model are consistent with recent research, showing that mice with the transgenic rearranged TCRs did not show age-related thymic involution [27] and suggesting that the transgenic α-chain expression could maintain normal TCR assembly and thymus functioning in aged mice. Our data are especially intriguing because a previous study has shown that β-chain rearrangements persist in the aged thymus [28].

Thymic involution reduces the population of naive cells that reconstitute a pool of peripheral T lymphocytes (Figure 2D, bottom panel) [29]. This is accompanied by compensatory clonal expansion of memory T cells, repopulating homeostatic niches [13,30]. These factors jointly contribute to the contraction of peripheral TCR repertoire diversity in the aged organism [13]. Accordingly, we observed significant TCR β-chain repertoire reshaping in old wild-type mice, but the peripheral TCR repertoire of 12-month-old TG mice was remarkably comparable to that of the young (3-month-old) TG mice (Figure 3). Notably, as the transgene was heterozygous in 1D1a mice, transgenic α-chain expression had no effect on the repertoire of endogenous TCR α-chains rearranged in the second allele (Figure 4). However, this analysis was restricted only to the Va families, for which commercial antibodies were available. Further precise studies, e.g., using NGS sequencing, are required to ascertain if heterozygous TCRα transgene expression could maintain the TCR repertoire diversity and normal thymus functioning in aged transgenic mice.

Dominant active TCRα expression affected the phenotype of CD8+ T cells in the spleens of intact (non-immunized) 3-month-old 1D1a mice, increasing the pool of T lymphocytes with the phenotype of central memory cells (Figure 2D, upper panel) [4]. Our recent studies have shown that TCR interaction with pMHC can influence T cell phenotype and function [4,17]. Strong competition for self-MHC-peptide complexes resulted in the accumulation of T cells with the naive phenotype, as seen in our TCRβ transgenic mouse model [17]. In contrast, transgenic α-chain expression can broaden TCR specificity, thus increasing the number of pMHC ligands available for recognition and forming a large pool of T lymphocytes with the phenotype of central memory cells as a consequence of TCR-pMHC interaction [4]. Importantly, the specific immune response was dramatically enhanced in transgenic 1D1a mice at all the studied ages. Our results demonstrated that 1D1a mice have an inborn pool of the antigen-specific T cells ready to immediately activate and accomplish their immune functions [4]. Thus, functional tests proved that the expression of the single dominant active TCR α-chain generated a pool of T cells with the phenotypic and, importantly, functional characteristics of true memory cells. Nonetheless, the response to third-party alloantigens was never compromised in 1D1a mice, indicating no contraction of the immune system’s reactivity because of transgenic dominant α-chain expression.

In 1D1a transgenic mice at all the studied ages, significant suppression of the humoral response was observed. Interestingly, similar to transient hypogammaglobulinemia in infant humans [31], decreased levels of IgM and IgG were registered in young (3-month-old) TG mice. This, however, could not be the sole reason for inhibition of the humoral response, as immunoglobulin levels in aged transgenic mice were restored to those in age-matched wild-type animals. Interestingly, Brändle et al. showed that the T cell help for antibody isotype switching was impaired in TCRα-transgenic mice [21]. In-depth evaluation of the T cell co-stimulatory activity and B cell functions in 1D1a mice will help to explain the effects observed in these studies.

To prove that autoreactive clones that could be potentially generated by the pairing of the transgenic dominant active α-chain with endogenous β-chains successfully eliminated by intrathymic negative selection in 1D1a mice, we performed histological evaluations of the lymphoid and non-lymphoid viscera of 1D1a mice in the age dynamics and found no pathomorphological changes in them. The data correlated well with our recent pre-clinical safety studies of the experimental 1D1α-modified T cell product [11]. The accumulation of 1D1α-transduced T cells in multiple organs of the recipient after adoptive transfer was not accompanied by damage to any of them, specifying no off-target activity or cross-reactivity of this dominant active TCR α-chain [11]. No blood test changes in 1D1a transgenic mice at all the ages studied further suggests normal systemic homeostasis in these animals.

Proteome blood analysis has become a powerful tool to monitor both the physiological and pathological processes in an organism [32]. To obtain a deeper insight into the systemic homeostasis of transgenic 1D1a mice that could be affected by dominant active TCRα expression, we performed comparative proteomics of the blood plasma of wild-type and transgenic mice. The analysis revealed sex- and age-dependent changes in the plasma proteomics of 1D1a mice. For both 1D1a females and males, alterations were found at the ages of 3 and 12 months, while any disturbances were absent in 6-month-old transgenic animals, indicating the restoration of normal homeostasis by this age. Interestingly, in young (3-month-old) and aged (12-month-old) transgenic mice (both female and male), altered proteins were not identical, suggesting random, but not systemic changes in their plasma proteome. Additionally, very few altered proteins were shared between young female and male transgenic mice. Among these, we found a 4–5-fold decreased plasma content of programmed cell death protein 6 (PDCD6), which is involved in TCR-, Fas-, and glucocorticoid-mediated apoptosis [33]. However, mice knock-out for PCDC6 showed no developmental abnormalities or immune disorders, and PCDC6 deficiency did not block apoptosis in T cells [34]. These data suggest that PCDC6 is functionally redundant and physiologically dispensable [34]. Accordingly, in the studies here, a drop in plasma PDCD6 levels had no adverse effects on the homeostasis and functioning of the immune system in young (3-month-old) transgenic 1D1a mice. Overall, the changes in plasma proteomics in transgenic mice revealed their stochastic and transient nature, with questionable physiological relevance. These changes cannot determine long-lasting adjustments in the immune system’s reactivity observed in 1D1a mice.

Taken together, our comprehensive studies here showed minimal and largely temporary changes in the immune system’s functioning and the overall systemic homeostasis in transgenic 1D1a mice that express the dominant active α-chain of the chain-centric TCR. These findings could point to differences in the ontogenies of wild-type and transgenic mice. The results presented here imply that plasma proteomics analysis is a potent approach for uncovering subtle, but possibly detrimental transgenesis-related changes in transgenic animals.

## 4. Materials and Methods

Animals. Wild-type B10.D2(R101) mice (K^d^I-A^d^I-E^d^D^b^) (WT), C57BL/6 (K^b^I-A^b^D^b^), and FVB (K^q^I-A^q^I-E^q^D^q^) mice were obtained from the breeding facility of the N.N. Blokhin National Medical Research Center of Oncology of the Ministry of Health of the Russian Federation (N.N. Blokhin NMRCO, Moscow, Russia). Transgenic 1D1a mice (TG) were generated as previously described [4]. Briefly, the full-length α-chain from 1D1 T cell hybridoma was cloned into the pTα cassette [20]. The single 1D1a line was established with the B10.D2(R101) genetic background in the Core Facility Center, Institute of Gene Biology, Russian Academy of Sciences (Moscow, Russia). The 1D1a mice were bred as heterozygous for the transgene in the Laboratory of Regulatory Mechanisms in Immunity, N.N. Blokhin NMRCO, by crossing with the parental B10.D2(R101) line. This allowed endogenous α-chains TCR rearrangements from the second allele, as TCRα rearrangement is not subject to allelic exclusion [12]. The genotyping of TG mice was performed as previously described [4]. TG mice were studied at the ages of 3, 6, and 12 months (3 Mo, 6 Mo, and 12 Mo, respectively), and age-matched WT mice of the parental B10.D2(R101) line were used as the control. If it is not otherwise stated, both female and male mice were used in studies here. Mice were housed in facilities maintained at 20–24 °C with a 40% relative humidity and a 12 h light/dark cycle. All mice had ad libitum access to standard rodent chow and filtered tap water. Mice were handled in strict compliance with the NIH guide for the care and use of laboratory animals (8th edition, 2011). All the experimental procedures were approved by the Ethical Committee on Animal Experimentation of N.N. Blokhin NMRCO. The experimental groups consisted of 3–12 mice. In acute experiments, animals were sacrificed by cervical dislocation.

Thymus and spleen cell isolation. The thymus and spleens from WT and TG mice of the studied age groups were aseptically isolated and individually homogenized in a Potter homogenizer in phosphate-buffered saline (PBS). Erythrocytes in the spleen cell suspensions were lysed in lysis buffer (BD Pharmingen, San Diego, CA, USA).Cell suspensions were then pelleted (200× *g* for 5 min at 4 °C) and resuspended in 3 mL PBS for flow cytometry analysis. Alternatively, splenocytes were resuspended in RPMI-1640 medium (PanEco, Moscow, Russia) supplemented with 10% fetal bovine serum (HyClone, GE Healthcare, Chicago, IL, USA), 0.01 mg/mL ciprofloxacin (KRKA, Novo mesto, Slovenia), 0.01 M HEPES (PanEco), and 10 mM 2-mercaptoethanol (Merck, Darmstadt, Germany) (complete RPMI) for an in vitro functional assay. Viable cells were counted after trypan blue/eosin staining in a Goryaev chamber.

Blood collection and analyses. Blood samples from WT and TG mice of the studied age groups were taken from the retro-orbital sinus. A blood test was immediately performed using a cell hematology analyzer (Nihon Kohden, Tokyo, Japan). To prepare plasma for subsequent analyses, blood samples were collected in individual 1.5-mL Eppendorf tubes coated with 0.5 M EDTA and centrifuged at 1500× *g* for 15 min at 4 °C. Plasma was then transferred to 0.5-mL Eppendorf tubes and stored at −80 °C before the proteome analysis and ELISA assays. To prepare serum, blood samples were collected in individual 1.5-mL Eppendorf tubes without EDTA, stored at 22 °C for 1 h, and centrifuged using the same regimen. The collected serum samples were immediately used for a biochemical blood test using the Cobas 501 analyzer (Roche Diagnostics, Rotkreuz, Switzerland) or for ELISA assays. Only plasma and serum samples without hemolyses were used in subsequent assays here. For flow cytometry analyses, blood samples were incubated with red blood cell (RBC) lysis buffer (eBioscience, San Diego, CA, USA) to remove erythrocytes, followed by two washes with PBS at 200× *g* for 5 min at 4 °C. Cells were then stained with fluorescent antibodies and analyzed using a flow cytometer (see below).

Antibodies. Cell samples were stained with the following antibodies: PE- or BV421-conjugated anti-CD3ε (clone 145-2C11, BioLegend, San Diego, CA, USA), APC-conjugated anti-CD8 (clone 53-6.7, BD Biosciences, San Jose, CA, USA), FITC- or Pacific blue-conjugated anti-CD4 (clone GK1.5, BD Biosciences), PE-conjugated anti-CD44 (clone IM7, BioLegend), APC-Cy7-conjugated anti-CD62L (clone MEL-14, BD Biosciences), FITC-conjugated anti-TCRγδ (clone GL3, BD Pharmingen), FITC-conjugated anti-CD19 (clone eBio1D3, eBioscience), APC-conjugated anti-Gr-1 (clone RB6-8C5, BioLegend), PE-Cy7-conjugated anti-CD11b (clone M1/70, BD Pharmingen), FITC-conjugated mouse Vβ TCR screening panel (cat. # 557004, BD Pharmingen), and FITC-conjugated anti-Va3.2 (clone RR3-16), anti-Va8.3 (clone B21.14), and anti-Va11.1/11.2 (clone RR8-1) (BD Biosciences).

Flow cytometry. Thymus and spleen cell samples (1.0–3.0 × 10^6^) were pre-incubated with Fc block (clone 2.4G2, BD Pharmingen) (10 min, 4 °C), and then stained with a mix of fluorescent antibodies (40 min, 4 °C). To characterize lymphocyte populations, 1.0 × 10^6^ events/sample were collected. For blood flow cytometry analyses, 0.5 × 10^6^ leukocytes were similarly stained with anti-CD3, CD4, CD8, Va3.2, Va8.3, and Va11.1/11.2 antibodies and the mouse Vβ TCR screening panel. To characterize the TCRα/β repertoire of peripheral T cells, 1.0 × 10^5^ events/sample were collected. The analysis was performed using the flow cytometer BD FACSCanto II (BD Bioscience) using the FACSDiva 6.0 software (BD Bioscience). For subpopulation characteristics, leukocytes were gated based on forward (FSC) and side scatter (SSC) parameters, followed by the gating of singlets by FSC-H vs. FSC-A plotting. Dead cells were excluded from the analysis by staining with propidium iodide (PI, BD Bioscience). The main thymocyte subpopulations were determined by the expression of CD4 and CD8 markers within the population of live singlets. For spleen cell analyses, live singlet cells were gated as described above. T cells were then gated by CD3 expression, followed by the gating of CD4+ and CD8+ cells. Subsequently, the co-expression of CD62L and CD44 markers was analyzed for CD3+CD8+ cells. The expression of TCRγδ was measured within the CD3+CD4−CD8− subset. B cells were gated by CD19 expression; monocytes/macrophages and neutrophils were gated as CD11b+ cells and Gr-1+CD11b+/− cells, respectively. In flow cytometry blood analysis, T cells were gated by CD3 expression, followed by the gating of CD4+ and CD8+ cells. The proportion of TCRVβ and TCRVα-expressing lymphocytes was estimated individually in the CD3+, CD3+CD4+, and CD3+CD8+ gates. Further processing of the data was performed using the FlowJo 7.6 software (TreeStar Inc., Ashland, OR, USA).

Pathomorphology. Thymus, spleens, lymph nodes, hearts, lungs, livers, kidneys, thyroid glands, pancreas, adrenal glands, and small and large intestines were isolated from WT and TG mice at all the ages studied. Each organ was fixed in 10% neutral formalin (JLS-Chemical, St. Petersburg, Russia), dehydrated in a gradient of alcohols, and embedded in Histomix (BioVitrum, St. Petersburg, Russia). Tissue sections (5 μm) were then prepared using an AccuCut SRM 200 microtome (Sakura, Alphen den Rijn, The Netherlands) and stained with hematoxylin and eosin. All samples were examined using an Eclipse E200 light microscope (Nikon, Melville, NY, USA). All evaluations were performed blinded to the animal group.

Mixed lymphocyte reaction (MLR). Spleen cells (3.0 × 10^5^ cells/well) from WT and TG mice were seeded in 96-well U-bottomed plates (Corning Costar, Sigma Aldrich, St. Louis, MO, USA) in 100 μL of complete RPMI in triplets. Spleen cells of C57BL/6 and FVB mice were used as specific and third-party stimulators, respectively. Spleen cells of B10.D2(R101) mice were used as the syngeneic stimulator to evaluate background cell proliferation. Stimulator splenocytes were treated with mitomycin C (Kyowa Hakko Kogyo Co., Ltd., Tokyo, Japan) (25 μg/mL, 37 °C, 30 min) and washed three times in PBS by centrifugation (200× *g*, 5 min, 4 °C). Stimulators (5.0 × 10^5^ cells/well) were then added to responder splenocytes in 100 μL of complete RPMI to the final volume of 200 μL, and cells were cultured at 37 °C with 5% CO_2_ for 72 h. Cell proliferation was measured by the incorporation of ^3^H-thymidine (Isotop, Moscow, Russia) added in the last 8 h of culturing. The level of cell proliferative activity is expressed as the number of counts per minute (cpm). To calculate the index of the antigen-induced immune response, the level of cell proliferation in the presence of stimulators (C57BL/6 or FVB) was divided by the level of the respective background proliferation.

Analyses of the humoral response. Female WT and TG mice of the studied age groups were intraperitoneally (i.p.) injected with sheep red blood cells (SRBCs) (ECOLab-Diagnostica, Moscow, Russia) at a dose of 5.0 × 10^7^ cells/mouse in 500 μL PBS. On Day 5, animals were sacrificed by cervical dislocation, and the number of plaque-forming cells in the spleen was assessed according to the N. Jerne method [35].

Evaluation of the blood Ig levels. The serum samples from non-immunized 3- and 6-month-old WT and TG female mice were collected as described above. IgM and IgG concentrations were evaluated by ELISA (Invitrogen, Thermo Fisher Scientific, Waltham, MA, USA).

Analyses of the delayed type hypersensitivity (DTH) reaction. Female WT and TG mice of the studied age groups were subcutaneously injected with 2 × 10^8^ SRBCs in 200 μL PBS. On Day 5, 10^8^ SRBCs in 50 μL PBS were injected into a hind limb paws of mice. An equal volume of PBS was injected into the contralateral paw. In 24 h, animals were sacrificed, and hind limb paws were cut at the ankle joint and weighted using laboratory scales, BM-512 (OKB Vesta, St. Petersburg, Russia). The index of the DTH reaction was calculated as follows:

$$\mathrm{DTH}\;\mathrm{index}\;\left(\%\right)=\frac{\mathrm{We}-\mathrm{Wc}}{\mathrm{Wc}\;}\times 100,$$

where We—weight of experimental paw; Wc—weight of control paw.

IFNγ and IL-4 production assays. Three-month-old female WT and TG mice were i.p. injected with SRBCs as described above. Three days later, mice were sacrificed, and spleen cell suspensions were prepared as described above. Cell samples (5.0 × 10^5^ cells/well) were seeded in triplicates in 96-well flat-bottomed plates (Corning Costar, Sigma Aldrich) in 200 μL complete RPMI. To induce cell activation, 3 μg/mL of concanavalin A (ConA) (Sigma Aldrich) was added to cells. Splenocytes were cultured for 24 h at 37 °C with 5% CO_2_. The culture supernatant of non-stimulated and ConA-stimulated cells was collected, and the levels of IFNγ and IL-4 production were then measured by ELISA (Thermo Fisher Scientific).

Preparation of plasma samples for proteome analysis. Plasma samples were collected as described above, individually from male and female WT and TG mice of all studied ages. Each sex and age group contained three animals. Reduction, alkylation, and digestion of the proteins were performed as described previously [36], with minor modifications. Briefly, 2 μL of plasma sample was mixed with 48 μL of sodium deoxycholate (SDC) reduction and alkylation buffer (pH 8.5) containing 100 mM TRIS, 1% (*w*/*v*) SDC, 10 mM tris(2-carboxyethyl)phosphine (TCEP), and 20 mM 2-chloroacetamide. The sample was heated at 95 °C for 10 min, cooled to room temperature, and an equal volume of trypsin/Lys-c mix (mass spec grade) (Promega, Madison, WI, USA) solution in 100 mM TRIS (pH 8.5) was added at a 1:50 (*w*/*w*) ratio. After overnight digestion at 37 °C, peptides were acidified by 40 μL of 2% trifluoroacetic acid (TFA) mixed with 80 μL of ethyl acetate and loaded onto SDB-RPS StageTips contained two 14-gauge SDB-RPS plugs. StageTip was centrifuged at 300× *g* until all the solution went through it (typically, 5 min). After 2 washings of the StageTips with 100 μL of 1% TFA/ethyl acetate (mix 1:1) and 100 μL of 0.2% TFA, peptides were eluted in a clean tube by 50 μL of 50% acetonitrile (ACN)/5% ammonia mixture by centrifugation at 300× *g*. The collected material was vacuum dried and stored at −80 °C. Before analysis, peptides were dissolved in 2% ACN/0.1% TFA buffer at a concentration of 0.5 μg/μL and sonicated for 1 min. To generate the spectral matching library, one plasma sample from a 3-month-old WT female mouse and one plasma sample from a 3-month-old TG male mouse were subjected to a procedure of enrichment of low-abundance plasma proteins with the ProteoMiner^tm^ enrichment kit (Bio-Rad Laboratories, Hercules, CA, USA) according to the manufacturer’s recommendations. Enriched low-abundance plasma proteins were then hydrolyzed, desalted, and dried as described above. The resultant tryptic hydrolysates were subsequently fractionated by SCX StageTips.

Fractionation of tryptic hydrolysates by SCX StageTips. For the SCX fractionation of tryptic hydrolysates, StageTips containing two 14-gauge SCX (3M, Saint Paul, MN, USA) plugs were used. SCX StageTips were conditioned with acetonitrile before the sample loading. The samples (40 μg) of the tryptic hydrolysate were diluted in 100 μL of 1% TFA and loaded onto the column. The column was sequentially washed with 0.2% TFA; a mix of 75 mM ammonium acetate, 20% ACN, 0.5% formic acid (FA); a mix of 125 mM _M_M ammonium acetate, 20% ACN, 0.5% FA; a mix of 200 mM ammonium acetate, 20% ACN, 0.5% FA; a mix of 300 mM ammonium acetate, 20% ACN, 0.5% FA; a mix of 600 mM ammonium acetate, 20% ACN, 0.5% FA; a mix of 5% NH_4_OH, 80% ACN, 0.5% FA. The resultant seven fractions were dried and reconstituted in the loading buffer as described above and individually analyzed by mass spectrometry.

Liquid chromatography and mass spectrometry. Samples were loaded onto a Acclaim PepMap 100 C18 (100 μm × 2 cm) trap column in the loading mobile phase (2% ACN, 98% H_2_O, 0.1% TFA) at 10 μL/min flow and separated at 40 °C using an Acclaim™ PepMap™ 100 C18 LC column (500 mm, 75 μm I.D., 2 μm particle size) (Thermo Fisher Scientific). Reverse-phase chromatography was performed with an Ultimate 3000 Nano LC System (Thermo Fisher Scientific) coupled to the Orbitrap Q Exactive HF mass spectrometer (Thermo Fisher Scientific) via a nanoelectrospray source (Thermo Fisher Scientific). Water containing 0.1% (*v*/*v*) FA was used as mobile phase A and ACN containing 0.1% FA (*v*/*v*), 20% (*v*/*v*) H_2_O was used as mobile phase B. Peptides were eluted from the trap column with a linear gradient: 3–35% solution B (0.1% (*v/v*) FA, 80% (*v*/*v*) ACN) for 105 min; 35–55% solution B for 18 min; 55–99% solution B for 0.1 min; 99% solution B for 10 min; 99–2% solution B for 0.1 min at a flow rate of 300 nl/min. After each gradient, the column was re-equilibrated with solution A for 10 min. MS data were collected in DDA mode (TopN = 15). The MS1 parameters were as follows: 120K resolution; 350–1400 scan range; max injection time—50 msec; AGC target—3 × 10^6^. Ions were isolated with a 1.2 *m/z* window, which is preferred for peptide match and isotope exclusion. Dynamic exclusion was set to 30 s. MS2 fragmentation was carried out at 15K resolution with an HCD collision energy of 28, a max injection time of 80 msec, and an AGC target of 1 × 10^5^. The other settings were as follows: the charge exclusion was unassigned from 1 to >7.

Data analysis. Raw spectra were processed using MaxQuant 1.5.3.30 [37] and Perseus [38]. The data were searched for in the *Mus musculus* Uniprot Tremble database, which contains canonical and isoform proteins (version from June 2016). The MaxQuant search was performed with the default parameter set, including Trypsin/p protease specificity, max 2 missed cleavages, met oxidation, protein N-term acetylation, and NQ deamidation as variable modifications, and Carbamidomethyl Cys as a fixed modification, max 5 modifications per peptide, 1% PSM and protein FDR. The following options were turned on: second peptide, maxLFQ, and match between runs. All runs were analyzed as independent experiments and processed in Perseus. In Perseus, the protein group results were filtered for contaminants, reverse, and “identified only by site” proteins. Only the proteins with a maxLFQ value in minimum of 3 out of 3 LC-MS runs in at least one group were used. For these proteins, missing values were imputed from the normal distribution with a 0.3 intensity distribution sigma width and 1.8 intensity distribution center that was downshifted. The two-sample *t*-test with Permutation based FDR of 5% was applied to search for significantly changing proteins. Mass spectrometry proteomics data were deposited in the ProteomeXchange Consortium via the PRIDE [39] partner repository with the dataset identifier PXD034188. Data were pair-wise compared for TG and WT females and TG and WT males of each age group. For statistical intergroup analysis, the paired Student t-test adjusted by the Benjamini–Hochberg procedure was used. An FDR value < 0.05 was considered to be significant. Gene set enrichment analysis was performed using the online resource DAVID [40,41]. Identified proteins with significant differences in the plasma content between each sex and age group of WT and TG mice were mapped onto the database KEGG (Kyoto Encyclopedia of Genes and Genomes) and sub-databases GOTERM_BP_DIRECT, GOTERM_CC_DIRECT, and GOTERM_MF_DIRECT. Data are presented as the ratio of the protein level in the blood plasma of TG mice to the respective level in the plasma of WT mice.

Statistical analysis. For all the parameters tested except for plasma proteomics, no sex-dependent differences were observed within the WT and TG groups at each age studied. Therefore, if it is not otherwise stated, the corresponding experimental data from both female and male mice were combined for statistical analysis and intergroup comparison. Comparative plasma proteome analyses were performed individually for male and female mice of each age group. Data are presented as mean ± SEM. All statistical analyses were performed using the Kruskal–Wallis test, the unpaired Student’s t-test, and the Mann–Whitney U-test using the Prism software (v. 8.1.2, GraphPad, San Diego, CA, USA). A *p*-value < 0.05 was considered to be significant.

## Data Availability

The mass spectrometry proteomics data have been deposited in the ProteomeXchange Consortium via the PRIDE partner repository with the dataset identifier PXD034188.

## Supplementary Materials

The supporting information can be downloaded at: https://www.mdpi.com/article/10.3390/ijms24076527/s1.

## Abbreviations

TCR—T cell receptor; TCRα/β—α/β-chain of a T cell receptor; MHC—major histocompatibility complex; pMHC—peptide-MHC complex; TG—transgenic mice; WT—wild-type mice; IFNγ—interferon γ; IL-4—interleukin 4; SRBC—sheep red blood cells; DTH—delayed-type hypersensitivity; ConA—concanavalin A; FA—formic acid; TFA—trifluoroacetic acid; ACN—acetonitrile; KEGG—Kyoto Encyclopedia of Genes and Genomes database; PDCD6—programmed cell death protein 6.
