# Supplementary material for: Physiological and Functional Effects of Dominant Active TCRα Expression in Transgenic Mice
Source: Int J Mol Sci. 2023 Mar 30;24(7):6527. doi: 10.3390/ijms24076527 (PMC10094918; doi:10.3390/ijms24076527)
Supplement: Supplementary file 1 [file ijms-24-06527-s001.zip › Supplementary Table S1.pdf]

Supplementary Table S1. Blood counts in transgenic 1D1a mice (TG) and wild-type B10.D2(R101) (WT) mice at the age of 3 - 12-months (mean ± SEM, n=5-7)

Parameter	Experimental groups					
	**WT**			**TG**		
	**3 Mo**	**6 Mo**	**12 Mo**	**3 Mo**	**6 Mo**	**12 Mo**
Leukocytes ($10^3/\mu L$)	11.5 ± 1.5	10.9 ± 1.3	11.5 ± 3.0	11.7 ± 1.8	8.2 ± 1.2	12.9 ± 2.9
Erythrocytes (RBC, $10^6/\mu L$)	8.9 ± 0.3	8.3 ± 1.4	7.9 ± 0.7	8.9 ± 0.8	8.6 ± 0.3	8.2 ± 0.9
Hemoglobin (HGB, g/dL)	137.0 ± 9.0	130.2 ± 4.2	116.3 ± 13.1	130.3 ± 7.8	124.7 ± 3.3	114.0 ± 21.4
Hematocrit (HCT, %)	35.8 ± 1.6	33.9 ± 5.2	33.0 ± 3.5	36.4 ± 3.7	35.2 ± 1.3	32.8 ± 4.2
Mean corpuscular volume (MCV, fL)	40.3 ± 0.9	41.1 ± 1.2	41.7 ± 1.9	40.9 ± 0.7	41.0 ± 2.0	40.1 ± 2.2
Mean corpuscular hemoglobin (MCH, pg)	15.4 ± 0.5	14.6 ± 0.3	14.7 ± 0.7	14.6 ± 0.6	14.5 ± 0.3	13.9 ± 1.8
Mean corpuscular hemoglobin concentration (MCHC, g/dL)	382.0 ± 13.2	355.8 ± 11.3	351.8 ± 14.9	359.0 ± 18.2	354.0 ± 9.5	346.0 ± 34.3
Platelets (PLT, $10^3/\mu L$)	710.7 ± 67.0	673.0 ± 113.1	850.0 ± 131.7	608.0 ± 71.3	721.2 ± 98.7	1045.8 ± 128.3
Red cell distribution width (RDW, %CV)	14.0 ± 1.9	12.9 ± 0.7	13.1 ± 0.7	13.3 ± 1.3	13.1 ± 0.9	13.9 ± 1.1
Thrombocrit (PCT, %)	0.3 ± 0.1	0.2 ± 0.06	0.3 ± 0.08	0.2 ± 0.02	0.2 ± 0.07	0.3 ± 0.1
Mean platelet volume (MPV, fL)	3.8 ± 0.3	3.5 ± 0.3	3.6 ± 0.5	3.2 ± 0.4	3.5 ± 0.4	3.8 ± 0.5
Platelet distribution width (PDW, %)	15.5 ± 1.5	15.2 ± 0.8	15.2 ± 0.9	16.1 ± 1.1	14.5 ± 0.8	15.1 ± 1.5
