# Supplementary material for: Physiological and Functional Effects of Dominant Active TCRα Expression in Transgenic Mice
Source: Int J Mol Sci. 2023 Mar 30;24(7):6527. doi: 10.3390/ijms24076527 (PMC10094918; doi:10.3390/ijms24076527)
Supplement: Supplementary file 1 [file ijms-24-06527-s001.zip › Supplementary Table S2.pdf]

**Supplementary Table S2.** Biochemical blood tests in transgenic 1D1a mice (TG) and wild-type B10.D2(R101) (WT) mice at the age of 3 - 12-months (mean ± SEM, n=3)

| Parameter | Experimental groups | | | | | |
|---|---|---|---|---|---|---|
| | **WT** | | | **TG** | | |
| | **3 Mo** | **6 Mo** | **12 Mo** | **3 Mo** | **6 Mo** | **12 Mo** |
| Total protein, mg/mL | 54.5 ± 0.6 | 54.9 ± 0.1 | 53.8 ± 1.0 | 52.7 ± 2.7 | 56.8 ± 0.2 | 53.0 ± 1.0 |
| Albumin, mg/mL | 31.0 ± 1.0 | 38.0 ± 0.3 | 33.0 ± 1.0 | 34.5 ± 1.5 | 37.0 ± 0.1 | 32.0 ± 4.0 |
| Urea, mmol/L | 7.9 ± 0.5 | 8.6 ± 0.3 | 7.3 ± 0.03 | 7.9 ± 0.4 | 9.2 ± 0.5 | 7.4 ± 0.5 |
| Creatinine, μmol/L | 19.0 ± 2.0 | 20.0 ± 1.0 | 15.0 ± 0.6 | 18.0 ± 3.0 | 18.0 ± 2.1 | 17.0 ± 0.01 |
| ALT, U/L | 36.1 ± 0.9 | 57.8 ± 2.4 | 85.0 ± 3.5 | 44.9 ± 11.7 | 51.7 ± 5.5 | 95.3 ± 11.4 |
| AST, U/L | 141.8 ± 19.8 | 84.9 ± 21.4 | 122.4 ± 20.7 | 156.3 ± 32.3 | 99.0 ± 32.2 | 167.8 ± 36.4 |
| Alkaline phosphatase, U/L | **108.5 ± 2.5** | 107.0 ± 2.4 | 61.7 ± 3.4 | **156.5 ± 10.6*** | 116.0 ± 1.4 | 74.5 ± 19.7 |
| Total cholesterol, mmol/L | 2.9 ± 0.2 | 2.2 ± 0.1 | 2.8 ± 0.1 | 3.1 ± 0.3 | 2.7 ± 0.3 | 2.2 ± 0.1 |

*p<0.05 compared to age-matched WT mice
