# Supplementary figures and images for: Physiological and Functional Effects of Dominant Active TCRα Expression in Transgenic Mice
Source: Int J Mol Sci. 2023 Mar 30;24(7):6527. doi: 10.3390/ijms24076527 (PMC10094918; doi:10.3390/ijms24076527)

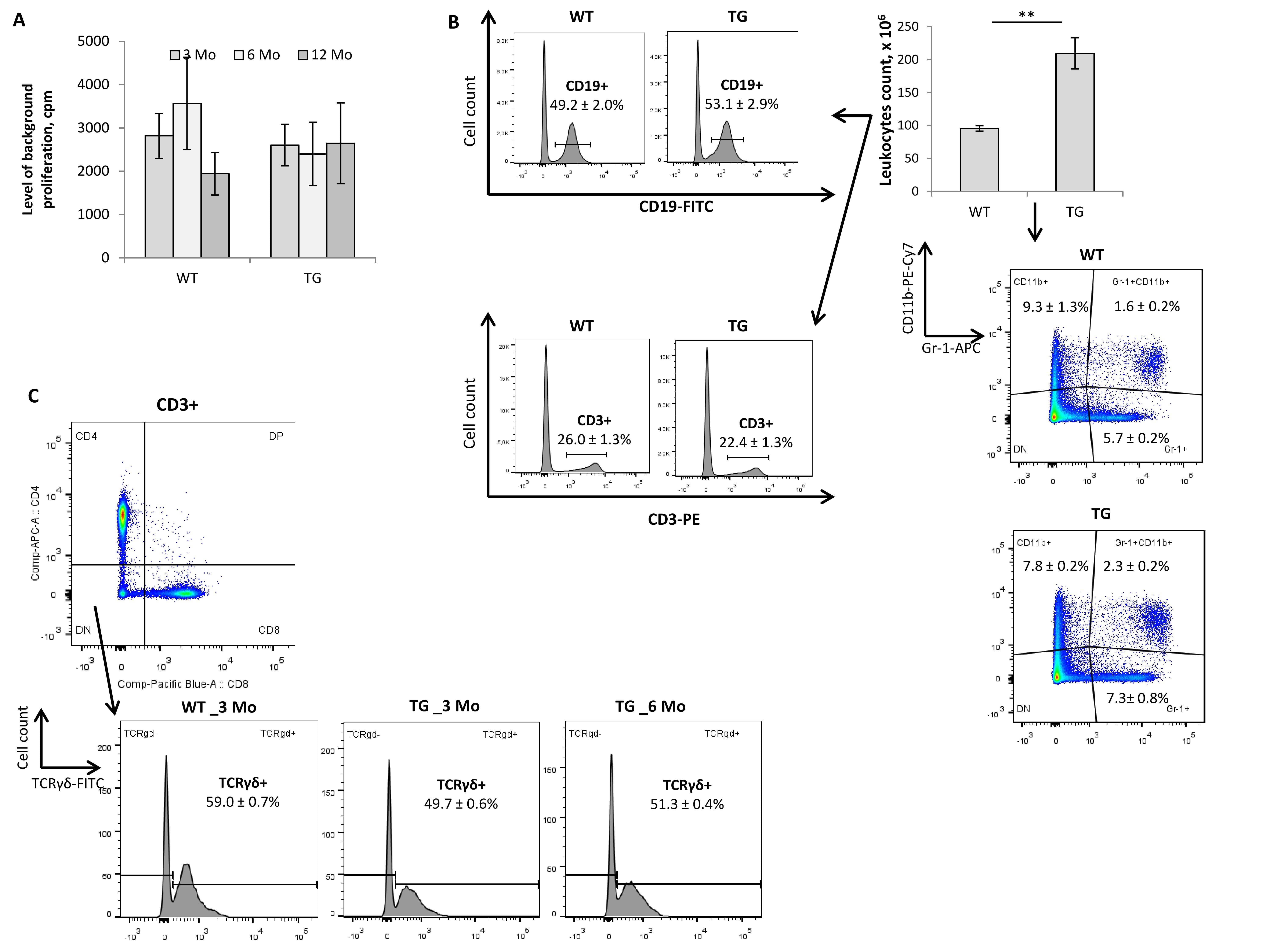

Supplement: Supplementary file 1 [file ijms-24-06527-s001.zip › Supplementary Figure S1.jpg]

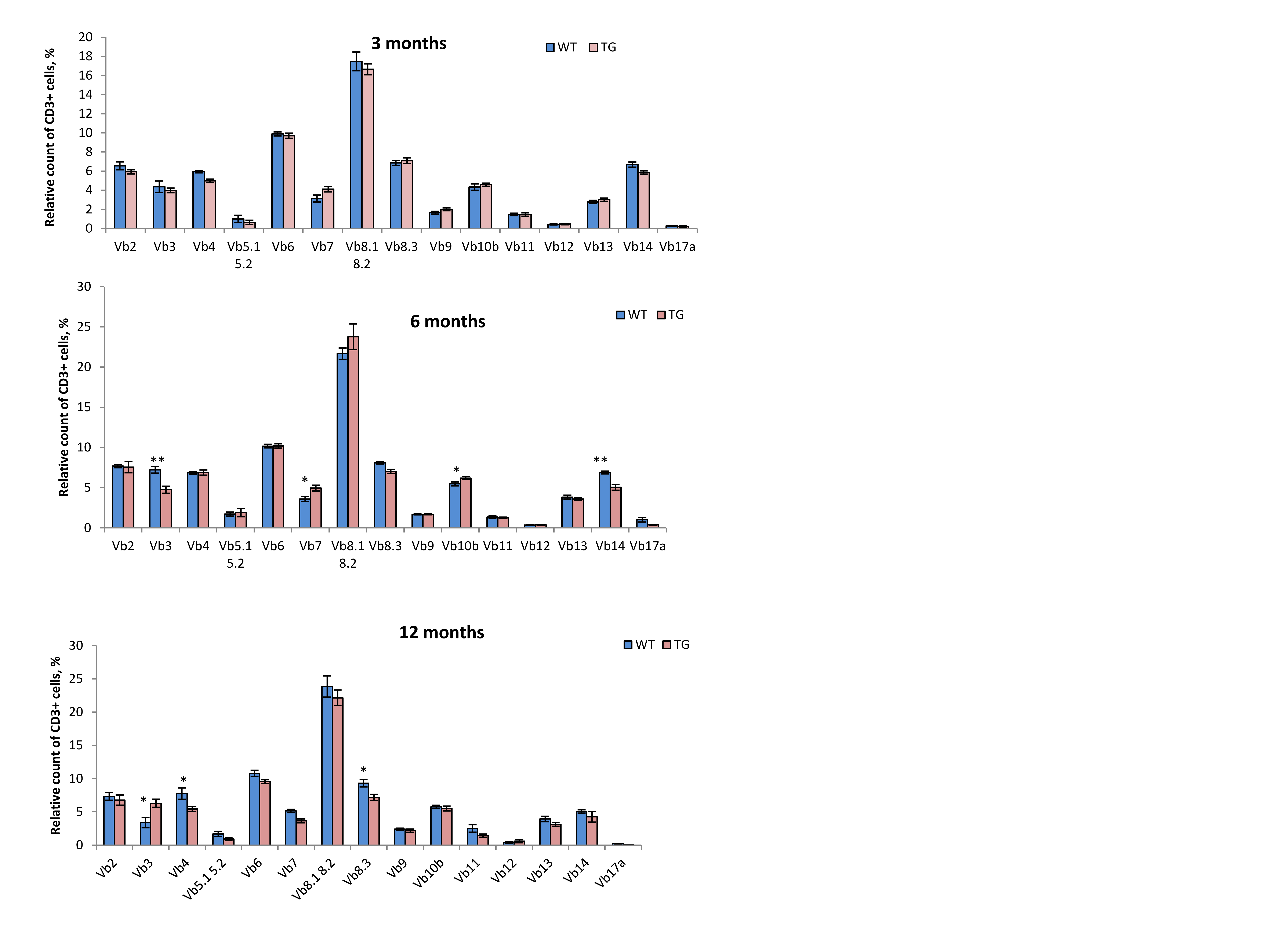

Supplement: Supplementary file 1 [file ijms-24-06527-s001.zip › Supplementary-Figure S2.jpeg]

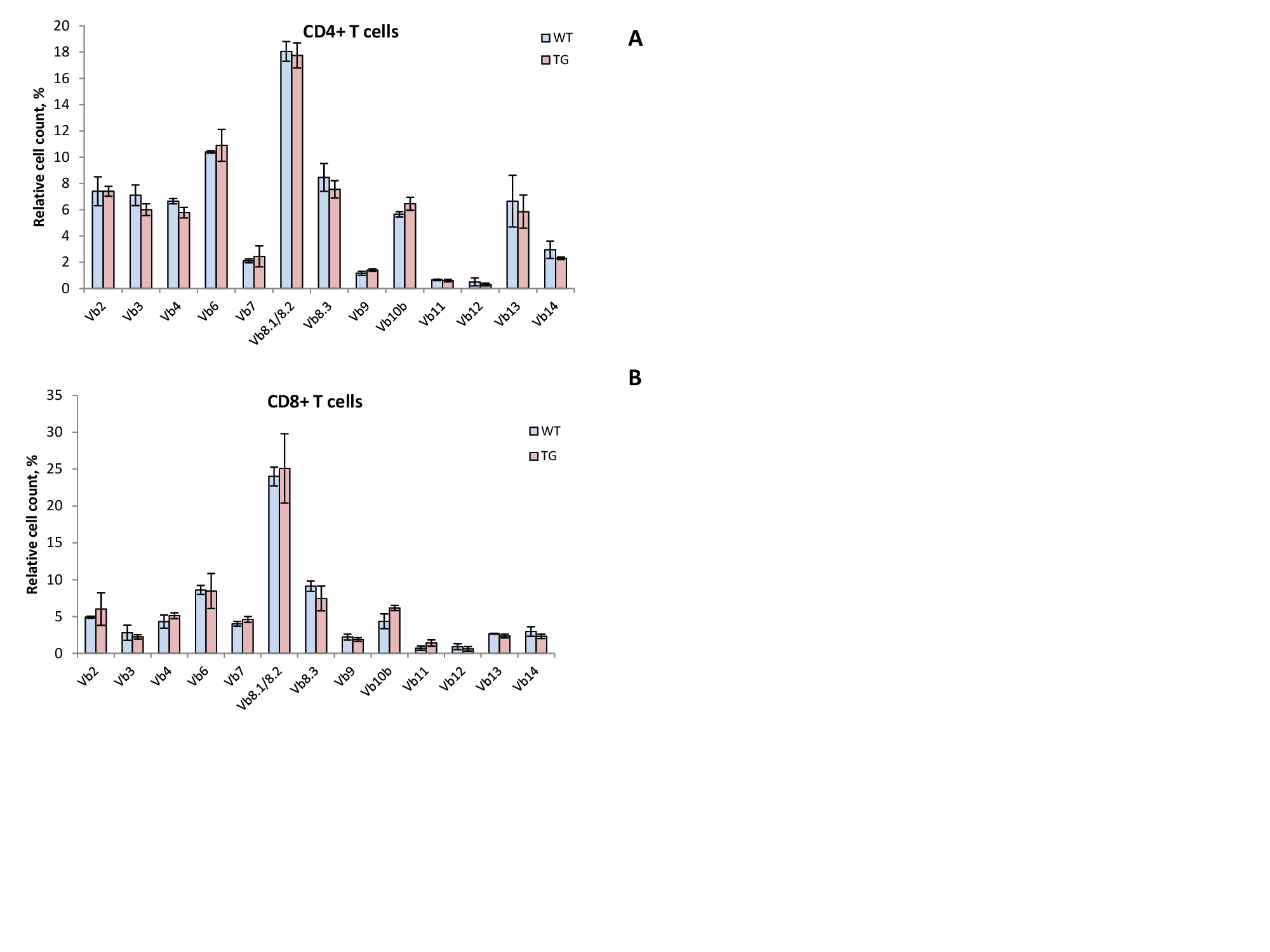

Supplement: Supplementary file 1 [file ijms-24-06527-s001.zip › Supplementary-Figure S3.jpeg]

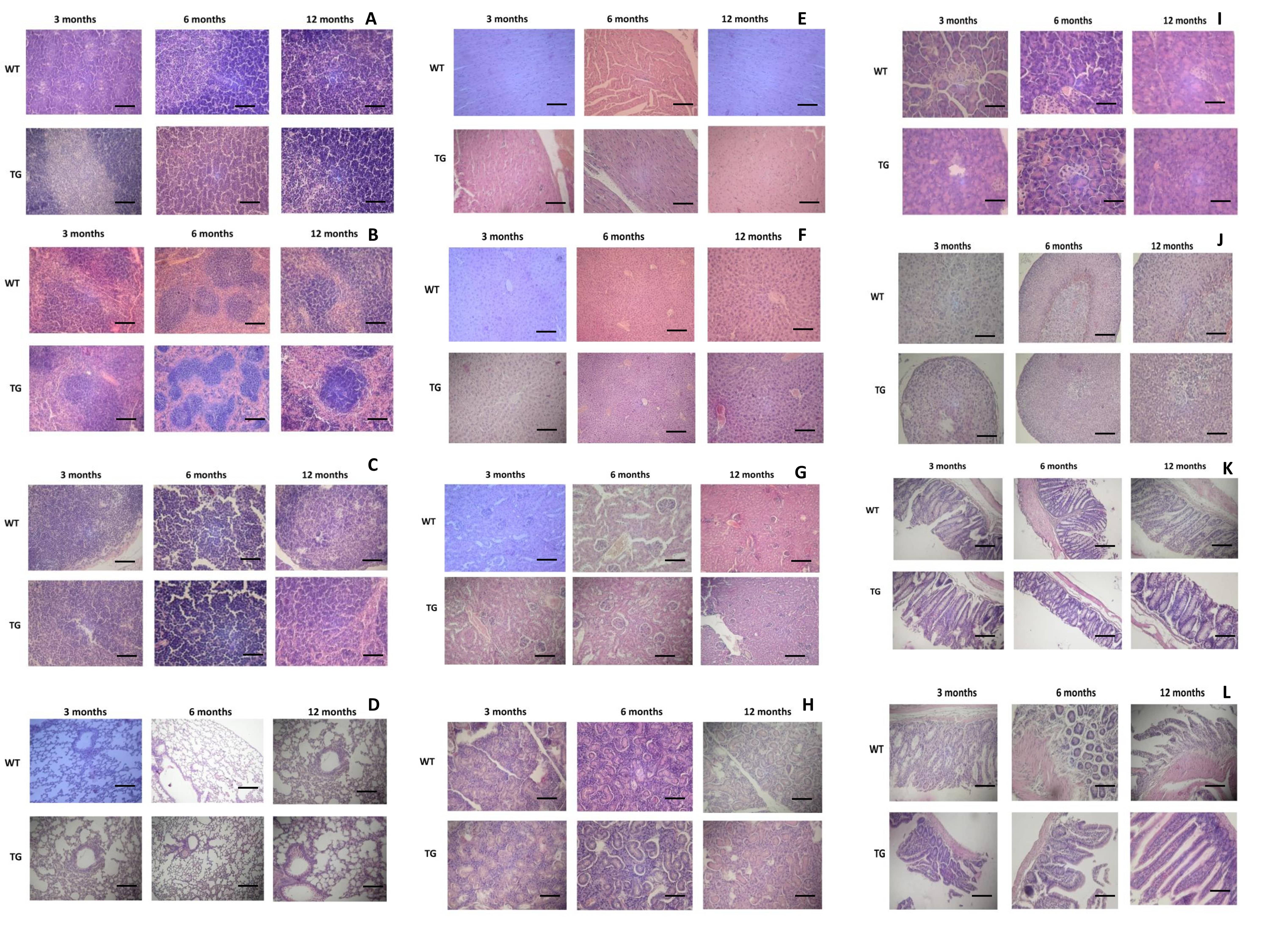

Supplement: Supplementary file 1 [file ijms-24-06527-s001.zip › Supplementary-Figure S4.jpg]

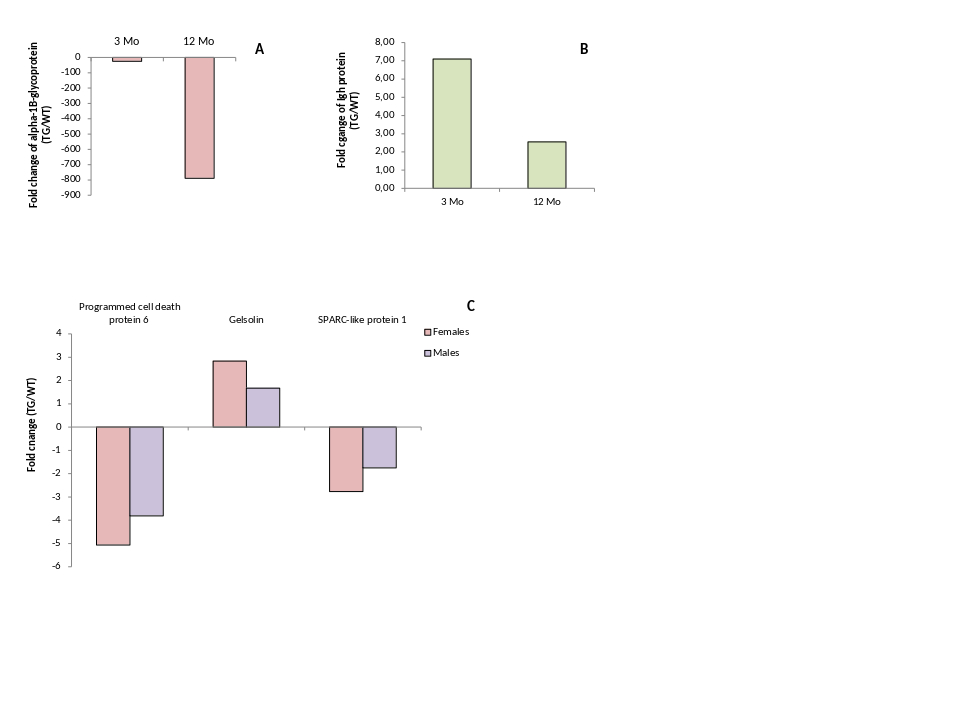

Supplement: Supplementary file 1 [file ijms-24-06527-s001.zip › Supplementary-Figure S5.jpeg]
